# Modeling individual differences in text reading fluency: a different pattern of predictors for typically developing and dyslexic readers

**DOI:** 10.3389/fpsyg.2014.01374

**Published:** 2014-11-18

**Authors:** Pierluigi Zoccolotti, Maria De Luca, Chiara V. Marinelli, Donatella Spinelli

**Affiliations:** ^1^Department of Psychology, Sapienza University of RomeRome, Italy; ^2^Neuropsychology Unit, IRCCS Fondazione Santa LuciaRome, Italy; ^3^Department of Human Movement Sciences and Health, University of Rome “Foro Italico”Rome, Italy

**Keywords:** reading, individual differences, dyslexia, suppression effect, RAN, vocal reaction times

## Abstract

This study was aimed at predicting individual differences in text reading fluency. The basic proposal included two factors, i.e., the ability to decode letter strings (measured by discrete pseudo-word reading) and integration of the various sub-components involved in reading (measured by Rapid Automatized Naming, RAN). Subsequently, a third factor was added to the model, i.e., naming of discrete digits. In order to use homogeneous measures, all contributing variables considered the entire processing of the item, including pronunciation time. The model, which was based on commonality analysis, was applied to data from a group of 43 typically developing readers (11- to 13-year-olds) and a group of 25 chronologically matched dyslexic children. In typically developing readers, both orthographic decoding and integration of reading sub-components contributed significantly to the overall prediction of text reading fluency. The model prediction was higher (from ca. 37 to 52% of the explained variance) when we included the naming of discrete digits variable, which had a suppressive effect on pseudo-word reading. In the dyslexic readers, the variance explained by the two-factor model was high (69%) and did not change when the third factor was added. The lack of a suppression effect was likely due to the prominent individual differences in poor orthographic decoding of the dyslexic children. Analyses on data from both groups of children were replicated by using patches of colors as stimuli (both in the RAN task and in the discrete naming task) obtaining similar results. We conclude that it is possible to predict much of the variance in text-reading fluency using basic processes, such as orthographic decoding and integration of reading sub-components, even without taking into consideration higher-order linguistic factors such as lexical, semantic and contextual abilities. The approach validity of using proximal vs. distal causes to predict reading fluency is discussed.

## Introduction

Fluent reading of texts is an important requisite for school achievement. The present study was aimed at investigating the factors that modulate individual differences in this skill.

Fluent reading aloud requires the integration of multiple sub-components (or process them in a cascaded manner according to the terminology adopted by Protopapas et al. (2013). When a word is being fixated and decoded readers plan the next saccade (based on para-foveal pre-processing of text on the right) but keep information about the previous words of the text so that they are able to utter them; readers also have to understand and memorize the meaning of what they are reading. A measure of this multiple-processing task is the asynchrony between eye position and speech output, referred to as eye-voice span (Buswell, 1921) or eye-voice lead (Fairbanks, 1937); indeed, typically developing readers are able to scan and process words much in advance of the word they are actually uttering.

In adult proficient readers reading aloud occurs fluently and effortlessly, with maximum reading speed for texts (in standard conditions) estimated at approximately 300 words per minute (Carver, 1982). Notably, even higher estimates are obtained using paradigms such as the rapid serial visual presentation which control for the influence of eye movements (e.g., Rubin and Turano, 1992). However, this performance is the endpoint of several years of practice, indicating slow power-function improvement in fluency (Zoccolotti et al., 2009). Notably, increases in reading speed (see data in Carver, 1982), as well as in the size of the eye-voice span (Buswell, 1921), have been observed up to college age.

Many children fail to acquire adequate reading skills, a deficit referred to as developmental dyslexia. Children with dyslexia do not learn to read fluently (e.g., Wimmer, 1993), produce frequent paralexias and characteristically have a very small eye-voice lead (e.g., De Luca et al., 2013). The literature on this disorder is large, particularly that focused on interpreting the nature of reading errors (e.g., see Castles et al., 2006; Temple, 2006; Friedmann and Lukov, 2008; Hulme and Snowling, 2014). Here we focus on the speed deficit of dyslexic children, that is, the deficit in reading fluency that is especially noted in languages with regular orthography (Wimmer, 1993; Zoccolotti et al., 1999). Considering reading fluency as the end-point of the integration of multiple sub-components of reading, some key questions arise. Which components contribute to the reading slowness shown by dyslexic children and how can they be measured and characterized? Does the need to integrate multiple sub-processes also contribute to generating the reading deficit?

To understand individual differences in reading fluency in typically developing and dyslexic children, we started from the working hypothesis that at least two basic factors contribute to the ability of all children to read fluently. The first is efficient orthographic analysis, i.e., *orthographic decoding*, and the second is the ability to integrate decoding of the on-going stimulus with utterance of the target and programming of the next saccade, i.e., *integration of reading sub-components*. The present preliminary study was aimed at evaluating whether these two components explain a relevant portion of the individual differences in text reading fluency. To rationalize our focusing on these two processes we capitalize on two major lines of reading speed research. The first one characterizes the basic difficulty in orthographic processing encountered by dyslexic children. The second one features studies that contrasted discrete and multiple presentations of stimuli and provides information on the integration of reading sub-components in typically developing and dyslexic readers. Below, we briefly review these two lines of research.

### Orthographic decoding deficits in dyslexia

A vast literature shows that orthographic decoding is the key difficulty in developmental dyslexia. Indeed, very clear reading deficits are detected in reading single words, i.e., when the requirement to read is stripped of the need to place the stimulus within a sentence and to pronounce it (e.g., van den Broeck and Geudens, 2012).

One related question is whether a developmental deficit can also be reliably detected for single letters or short letter strings. It is generally held that children with dyslexia show deficits in reading words (e.g., Katz and Wicklund, 1971) but not in recognizing letters (e.g., Katz and Wicklund, 1972). Notably, this sparing has also been shown with methodologies that allow controlling for the general difficulty of the task. For example, Martelli et al. (2009) examined the contrast threshold to identify single letters and words and found that dyslexic and typically developing readers needed about the same amount of contrast to identify single letters but differed greatly in the case of long words. Bosse et al. (2007) found that dyslexic children were not impaired in identifying briefly presented letters but had severely impaired visual spans, i.e., they were unable to process a multi-element array of consonants in parallel. In a later study (Lassus-Sangosse et al., 2008), they showed that the string letter deficit was present only when the presentation of letters was simultaneous not when it was sequential. In a similar vein, De Luca et al. (2010) found that dyslexic children were only mildly affected in letter, bigram and two-letter syllable tasks but were severely affected in the case of both words and non-words. Performance in these latter tasks was well accounted for by a single global factor referred to as a “*letter-string*” factor to mark, on one hand, that it was present only in the case of multi-letter displays and, on the other, that it was independent from lexical activation.

The presence of this global letter-string factor has been confirmed in a number of studies that provide information about its characteristics (Zoccolotti et al., 2008; Paizi et al., 2011, 2013; Di Filippo and Zoccolotti, 2012). In particular, the global factor that marks the decoding deficit of children with dyslexia was present when they named orthographic but not pictorial stimuli (Zoccolotti et al., 2008) and when targets were presented visually but not acoustically (Marinelli et al., 2011). Notably, in all these studies the global factor accounted for a very large proportion of the variance in group differences between dyslexic and typically developing readers. Overall, children with dyslexia are severely impaired in decoding when the task requires the parallel processing of a string of letters presented visually regardless of whether the letter string represents a legal word or not. We proposed that this global factor indicates a deficit in a pre-lexical “grapheme description” independent of case, font, location or orientation (see Marsh and Hillis, 2005). Dehaene et al. (2005) proposed a neural model to account for the abstract ability to process words regardless of their location, font and size. According to the Local Combination Detector (LCD) model written words are encoded by a hierarchy of detectors tuned to increasingly larger and more complex word fragments (visual features, single letters, bigrams, quadrigrams and, possibly, words). Over years of practice, learning of local combination detectors allows portions of the left ventral occipito-temporal visual system (referred to as visual word form area, VWFA) to become attuned to the regularities of the writing system, yielding fast parallel processing in reading (Cohen et al., 2000, 2002). The construction of this mechanism seems defective in dyslexic children (Richlan et al., 2009; Pontillo et al., 2014). This mechanism fits well with the pre-lexical “grapheme description” we found defective in dyslexic children.

In the cited studies of dyslexic children (i.e., Zoccolotti et al., 2008; Marinelli et al., 2011; Paizi et al., 2011, 2013; Di Filippo and Zoccolotti, 2012), in agreement with the predictions of the rate and amount model (RAM, Faust et al., 1999) the presence of a letter-string factor was inferred through linear regression analysis on the basis of performance on a large variety of tasks (reading high- or low-frequency words of different lengths, making lexical decisions on words or pseudo-words, etc). Notably, the predictions of the RAM apply at both a group and individual level (Faust et al., 1999). Thus, one may use the parameters of the linear regression of the condition means of a given dyslexic child over those of the total group of readers to obtain estimates of the impairment of the child in terms of the global factor (for a discussion on this point see Kail and Salthouse, 1994). For example, van den Boer et al. (2013) recently showed that the slope and the intercept were expressing different reading processes: the slope indicated the degree of serial processing while the intercept expressed the overall reading speed of words and non-words. Based on the RAM, individual slopes calculated for reading words and pseudo-words using RTs (De Luca et al., 2010) or mean total reading times per item (Di Filippo and Zoccolotti, 2012) correlated significantly with reading speed (and accuracy) in a standard reading test.

However, when studying reading with a correlational approach as in the present study, the use of a single target task may prove advantageous to establish individual performance as compared to the extraction of a single index from a variety of experimental conditions. On the one hand, it is considerably more economical. On the other hand, it avoids the difficulty of obtaining reliable regression coefficients (i.e., slopes and intercepts) at an individual level. Indeed, these are typically based on relatively few conditions and few trials per condition on each observer; thus, individual outliers may occasionally be present for whom the linear regression accounts for only a small proportion of variance. As described in greater detail below, in the present study, we selected a task particularly apt to measure orthographic decoding ability i.e., reading visually displayed single pseudo-words with the instruction to read as fast as possible (ASAP). This task captures the critical characteristics of the letter-string factor because it is in the visual modality and it calls for the fast parallel processing of a string of graphemes without requiring direct access to the lexicon. At the same time, it does not imply the ability to deal with multiple items as this represents a separate factor contributing to reading fluency. Note that processing of a letter string requires dealing with multiple elements (i.e., a set of graphemes) in parallel. Thus, if parallel processing for string is not developed, such as when learning to read, integration processes are evident also within a single word, and, for example, this is indicated by multiple fixations on the string and/or parceled uttering of the target. In the present context with 6th graders, we only focus on the contrast between the orthographic decoding of a single (although in itself complex) target with the ability to integrate this processing with the decoding of other adjacent targets as typical of functional reading.

### Integration of reading sub-components: discrete- vs. multiple-stimulus presentation

Fluent reading requires the ability to integrate the decoding of the on-going stimulus with utterance of the target and programming of the next saccade. This ability implies various sub-components. Previous research has shown that sub-components, such as visual scanning or eye movements, are not affected *per se* in dyslexic children. Thus, scanning and eye movements appear largely unimpaired if non-linguistic stimuli are presented (e.g., Brown et al., 1983; Olson et al., 1983; De Luca et al., 1999). Similarly, no articulatory deficit is present (e.g., Di Filippo et al., 2005; Wimmer et al., 1998).

However, there is evidence suggesting that *integration of the subcomponents involved in reading* is defective in children with dyslexia also when they perform a non-orthographic task. This evidence comes from studies comparing the presentation of discrete- vs. multiple-stimulus displays. Indeed, several of these studies stemmed from research on the paradigm known as “rapid automatized naming” or RAN (Denckla and Rudel, 1974, 1976). In the typical display, the child has to name 50 stimuli (i.e., digits, patches of colors, drawings of objects, etc.) regularly placed on a sheet of paper. Only a few targets (usually five) are used for each trial. The children are trained so they have no uncertainty about the repeated target names. Denckla and Rudel (1976) reported that dyslexic children performed this task more slowly than typically developing readers but were relatively accurate. The nature of the dyslexic children's difficulty in this seemingly simple task has been debated.

Some authors see RAN as just another example of a phonologically laden task (e.g., Ramus et al., 2003). In this view, dyslexic children are slow because of their inefficiency in retrieving the color, digit or picture names. Some correlational evidence goes in this direction. Thus, performance on RAN tasks generally correlates with performance on other phonological awareness tasks (Katz, 1986; Wagner and Torgesen, 1987; Compton et al., 2001; Chiappe et al., 2002). An alternative interpretation was advanced by Wolf and Bowers (1999; see also Wolf et al., 2000). They proposed that RAN is highly correlated with reading as it reproduces its “*microcosm*,” i.e., it involves all the sub-components comprising functional reading with the exception of orthographic decoding (see also Blachman, 1984). In this view, dyslexic children are impaired because they are slow in organizing a fluent stream of multiple processes. In this hypothesis, the comparison between discrete and multiple presentations of stimuli is crucial, as only the latter format should show a relationship with reading. By contrast, according to a phonological explanation inefficiency in retrieving color, digit or picture names is expected in both cases. Supporting Wolf and Bowers's view, much research has shown that if stimuli (i.e., digits, colors, pictures) are presented individually, correlations with reading skills are lower than with serial naming (e.g., Stanovich et al., 1983; Bowers, 1995; Chiappe et al., 2002; Logan et al., 2011).

Several studies have dealt with this issue in the last few years. de Jong (2011) examined the development of the relationship between RAN and reading fluency as a function of the format (i.e., discrete vs. serial stimulus presentation) in first, second and fourth grade children. The author found that similar formats of RAN and reading were more strongly related than dissimilar formats among “advanced readers” (i.e., children that read words by sight; almost all 2nd and 4th grade children). Discrete RAN was more related to discrete reading fluency of high-frequent one-syllable words than with the serial reading of these words, while serial RAN was more related to serial words reading fluency than with discrete word reading. Moreover, discrete RAN made a unique contribution in predicting discrete word reading among “advanced readers,” whereas serial RAN did not. On the contrary, for “beginning readers” (i.e., those who still read such words serially), RAN was the strongest predictor (whereas the contribution of discrete RAN was negligible) in word reading irrespective of the serial-discrete format (see also de Jong, 2008). Note that serial RAN predicted a large amount of unique variance in serial word reading in both advanced and beginning readers. In a recent study, Georgiou et al. (2013) compared discrete and serial RAN in a variety of experimental conditions. They found that RAN was related to reading partly because it involved serial processing (no correlation with reading was present in the case of discrete naming) and partly because it required the oral production of the different names of the stimuli. In fact, the correlation with reading dropped when subjects were instructed to give fixed oral responses to target and non-target stimuli (i.e., yes or no, 2 or 5, and apple or chicken). Georgiou et al.'s (2013) findings indicate that the whole set of cognitive operations involved in reading is necessary to yield the relationship between RAN tasks and reading. In the same vein, it has been observed that scanning the same RAN targets to cross out a given target is not correlated with reading (see also Wimmer et al., 1998; Landerl, 2001; Di Filippo et al., 2005; Georgiou et al., 2013).

Logan et al. (2011) found that serial naming uniquely predicted reading and that the relation was stronger when isolated naming was controlled for, suggesting that isolated naming functioned as a suppressor variable in the relation between serial naming and reading. In the case of suppression an independent variable contributes little or no variance to the dependent variable but may have a sizeable beta weight because it “purifies” one or more independent variables of their irrelevant variance, thereby allowing their predictive power to increase (Capraro and Capraro, 2001). Notably, specific analyses are needed to show these suppression effects (typically not adopted in early research on serial-discrete RAN). Evidence for a suppressive effect was recently confirmed by Protopapas et al. (2013) who compared the performance on discrete and serial naming of digits, objects, and words of second and sixth grade Greek children. Discrete and serial word reading correlated highly in younger children but less in older children. A reading–naming dimension explained the data well for the younger children; by contrast, a dimension in terms serial-discrete processing emerged with older children. Thus, although RAN and reading are correlated at different ages the underlying structure of this relationship may actually change as a function of reading experience. So, younger children appeared to process stimuli predominantly as a series of isolated items while older children start using serial procedures in a cascaded manner effectively. Protopapas et al. (2013) also examined the contribution of naming tasks over and above that of the effect of discrete word reading through regression and communality analyses. The results for communality analyses are particularly relevant here as we used the same approach. For sixth graders, multiple RAN contributed unique variance to the prediction of serial words, while discrete word reading was a minor contributor. The reverse held for younger children; in this case there was a large contribution of discrete word reading and multiple RAN did not explain unique variance.

Notably, most research on discrete and multiple targets is correlational and direct experimental comparisons between these two types of presentation are very few (particularly in the case of reading tasks). One possible reason is that different (and not directly comparable) dependent measures are characteristically used in the two domains. Studying the reading of isolated words (and non-words) largely rests on the analysis of vocal reaction times (RT). Thus, only the time between stimulus onset and the beginning of the vocal response is measured; this putatively captures the decoding part of the response, whereas the actual pronunciation is usually considered as not interesting (but, for a recent analysis of the characteristics of the pronunciation component of the response see Davies et al., 2013; Martelli et al., 2014). By contrast, reading fluency with multiple stimuli, such as word lists or texts, is measured by calculating the time needed to entirely process each stimulus. Thus, the whole time needed to decode and utter a target is considered in this case. Analysing total reading time of discrete stimuli (i.e., the time from onset of the stimulus to the end of the pronunciation) allows for a direct comparison between reading of discrete vs. multiple words (or non-words).

Using this approach, we recently found that 12 years-old typically developing readers had a clear advantage on multiple over discrete items in both RAN and reading tasks (Zoccolotti et al., 2013). Thus, they were able to partially process the next visual stimulus while uttering the current target, producing the time advantage over discrete items. The children with dyslexia of the same age showed a smaller advantage for multiple stimuli in naming colors and digits but presented the opposite pattern in reading, i.e., they were faster when they read discrete than multiple targets. Accordingly, we proposed that dyslexic children's great impairment on multiple arrays indicates a selective difficulty in integrating the multiple subcomponents of the reading task (Zoccolotti et al., 2013). As stated above, direct comparisons of reading under discrete and serial conditions are lacking; thus, to the best of our knowledge, we cannot compare our data with those of other laboratories. Using a somewhat different paradigm, Jones et al. (2009) directly compared discrete and multiple RAN-type tasks and reported that dyslexic young adults showed a greater deficit for multiple than discrete items, whereas non-dyslexic individuals showed a marginal facilitation with this format.

Overall, it seems that the integration of multiple subcomponents (analogous to those implied in reading) is defective in dyslexic children over and above the basic nuclear deficit in decoding words (Zoccolotti et al., 2013). Thus, in the present study, we considered integration ability as a separate factor in predicting reading fluency.

### Present study

The present study aimed to evaluate the factors that account for individual differences in the reading fluency of typically developing and dyslexic readers. As dependent measure we chose to examine reading of texts rather than single words because it has a clear functional value and includes dealing with both orthographic materials and multiple target displays. These two latter aspects correspond to the two critical factors we selected to account for children's ability to read fluently: (1) decoding strings of letters presented visually (referred to as *orthographic decoding)*; and (2) integrating decoding of the on-going stimulus with utterance of the target and motor preparation of the next saccade, which requires parafoveal analysis of the future target (referred to as *integration of reading sub-components)*. Both factors are active when children read a meaningful text. However, measuring reading fluency does not directly allow understanding which of them is responsible (and to what extent) for a reading delay because both factors are involved in the performance. Indeed, only one of them (or both but to a variable degree) may be inefficient. A model that separately evaluates the contribution of these two factors may offer, at least in principle, the basis for future investigations of selective disturbances of each factor and/or their interaction.

To measure these two factors separately, we selected single pseudo-word reading and a standard RAN task requiring the naming of digits (or colors). As stated above, single pseudo-word reading appears as a particularly appropriate measure of the ability to decode a string of letters. Critically, on one hand, this performance does not require integrating multiple subcomponents (as in standard reading) and on the other hand it does not involve the orthographic lexicon. The performance of digit (or color) RAN represents a particularly suited measure of the ability to integrate the various sub-components typically involved in reading except for orthographic decoding (and keeping lexical and semantic processing aside).

This proposal may be seen as a simplified schema of the processes involved in text reading fluency. As proposed above, the motivation to develop this model stems from the observation that dyslexic children's impairment on multiple stimuli cannot be entirely explained by their single word performance (Zoccolotti et al., 2013). Although they have many different key features, most accepted models of reading, such as the dual route model (Coltheart et al., 2001), the CDP+ model (Perry et al., 2007) or the triangle model (Plaut et al., 1996), focus on the word level; thus, they are only partially informative when examining dyslexic children's reading slowness on texts and more generally when the aim is to predict reading fluency.

Clearly, the proposed model is only a skeleton focused on the processes that, based on previous research, we expect to be closely related to individual differences in text reading fluency. A full model would require specifying all the processes involved in reading fluency (e.g., spelling out all the processes that converge to determine the “integration of the reading sub-components” factor); this enterprise is beyond the aims of the present study which was intended as a first step in this direction. At any rate, it is important to keep in mind that other factors may also play a role in predicting individual differences in text reading fluency. In particular, higher-order linguistic factors may moderate this relationship. These in turn should include efficiency in accessing the orthographic and the phonological lexicon as well as semantic and contextual abilities. In this present preliminary study, however, we were specifically interested in examining how much individual differences in reading fluency can be accounted for by relying only on basic reading processes.

One question concerns the relative independence of the two factors considered. For instance, to explain dyslexics' difficulties in RAN tasks Wolf et al. (2000) proposed that there are “*connections among processes underlying naming speed, automatic orthographic pattern recognition, word identification, and reading fluency*” (Wolf et al., 2000). According to this “connection” hypothesis, one would expect the two factors to be partially related in their influence on reading fluency.

Operationally, we tested whether two variables (discrete pseudo-word reading and multiple RAN) alone or in combination significantly predicted reading fluency on meaningful texts. For RAN, both digit and color stimuli were used. It has been proposed that these two sets of stimuli generate partially different patterns of response (e.g., van den Bos et al., 2002). Notably, naming digits requires the arbitrary mapping of visual stimuli into phonological labels and is expected to produce generally more automatic processing; naming colors is mediated by semantic activation and yields generally slower and less automatized responses than digit stimuli. Thus, we decided to analyze digit and color conditions separately. As a measure, we considered a unit (i.e., total reading time per item) that was directly comparable with both discrete and multiple stimulus presentations as well as reading and naming tasks. We expected both variables i.e., discrete pseudo-word reading and multiple RAN, to contribute unique variance to the prediction and evaluated whether they also shared a common portion of the variance. Moreover, we used an additional control task, i.e., naming times for the isolated presentation of digits (or colors) which, based on previous research, was expected to contribute to the variance indirectly by acting as a suppressor variable (Logan et al., 2011; Protopapas et al., 2013; Logan and Schatschneider, 2014). As we expected predictors to show varying degrees of inter-correlation we used commonality analysis, a type of multiple linear regression that allows partitioning the total variance explained by independent variables into variance unique to each variable and variance shared by a subset of independent variables (Pedhazur, 1982). Commonality analysis is particularly suited when collinearity of predictors is expected as well as the presence of suppression effects (Nimon and Reio, 2011). Based on previous research, we expected a suppression effect of the discrete naming variable (Logan et al., 2011; Protopapas et al., 2013).

First, we present data relative to a group of typically developing readers (Study 1); second, we present data relative to a group of dyslexic readers, highlighting possible differences in the weight of predictors between the two groups (Study 2). In the main text we report data using the digit conditions (both RAN and discrete naming); we synthetically report the same analyses for the color conditions for both typically developing and dyslexic children as Supplementary Materials.

## Study 1: predicting reading fluency in typically developing readers

Below we present data from a group of 11- to 13-year-old children with typical reading development. At this age level acquisition of reading speed is almost complete (Zoccolotti et al., 2009). Furthermore, recent evidence indicates that in children in this age range the processing of multiple displays is well differentiated from that of isolated stimuli (Protopapas et al., 2013).

### Materials and methods

#### Participants

Forty-three typically developing readers (20 males and 23 females; mean age 11.6 ± 0.4 years) participated in the experiment. Non-verbal IQ level was assessed using Raven's Colored Progressive Matrices. All children scored well within the normal limits according to the Italian norms (Pruneti et al., 1996); mean raw score was 28.8 ± 3.4; mean z score was −0.32 ± 0.80. Reading efficiency was assessed by the MT Reading test (Cornoldi and Colpo, 1995, see below). All participants had normal or corrected-to-normal visual acuity.

#### MT reading test

The child reads a passage aloud within a 4-min time limit. Reading time (s/syllable) and accuracy (number of errors, adjusted for the amount of text read) are scored (Cornoldi and Colpo, 1995). As for raw data, the average reading time per syllable was 0.23 s (*SD* = 0.04), and the mean number of errors was 6.2 (*SD* = 3.6). Mean z scores (based on normative values, Cornoldi and Colpo, 1995) were near zero for all parameters (0.02 and −0.09 for reading time and accuracy, respectively).

Note that, for the specific aims of the present study, the reading speed at the MT test was the dependent measure for estimating text reading fluency. As for all other measures (see below), an inverse transformation was applied to the data so that item/s was considered in the statistical analyses.

#### Reading pseudo-words

Twenty 5- and 20 7-letter pseudo-words (matched for initial phoneme across lengths) were derived from words by changing one (or two) letter(s) of each word (see Appendix). Words were selected from the LEXVAR database (Barca et al., 2002; http://www.istc.cnr.it/grouppage/lexvar) and were matched for frequency across length (mean log frequency = 1.4) as well as for bigram frequency (according to the children corpus of word frequency by Marconi et al., 1993). The mean number of syllables was 2.0 for five-letter items and 2.9 for seven-letter items.

Pseudo-words appeared in black lowercase Times New Roman on a white background. Center-to-center letter distance subtended 0.4° horizontally at a viewing distance of 57 cm. Items were singly presented on a PC screen in two blocks, separately for the two lengths.

#### Naming digits and colors

Stimuli were five digits (2, 4, 6, 7, and 9) and five colored squares (black, yellow, and the primary green, red, and blue, digitally defined according to the red, green and blue (RGB) triplets for standard colors) on a white background. Both digits and color names had a mean number of syllables of 1.8 (mean of letter length = 4.6 for colors and 4.4 for digits, respectively) and did not differ for bigram frequency (Marconi et al., 1993). Note that pseudo-words in the reading experiment did not differ from digit names for bigram frequency (Mann-Whitney *U* Test, *Z* = −0.28, n.s.), but differed for number of syllables (Mann-Whitney *U* Test, *Z* = 6.18, *p* < 0.0001) and letters (Mann-Whitney *U* Test, *Z* = 5.54, *p* < 0.0001); pseudo-words differed from color names for bigram frequency (Mann-Whitney *U* Test, *Z* = −4.06, *p* < 0.0001), number of syllables (Mann-Whitney *U* Test, *Z* = 5.17, *p* < 0.0001) and number of letters (Mann-Whitney *U* Test, *Z* = 5.54, *p* < 0.0001).

In the discrete stimulus condition, a single digit (color) appeared in the screen. Twenty-five digit- and 25 color-trials were given in two separate blocks. In the multiple stimuli condition (RAN), 100 digits and 100 colored squares were printed on separate sheets of A4 paper; there were two sheets for each stimulus type (for a total of four sheets), each containing an array of 50 items arranged in 10 rows of five columns.

Each digit (Helvetica, black) subtended 0.9° and each square 2.5°, horizontally, both in the discrete (at 57 cm viewing distance) and the multiple (at 40 cm viewing distance) conditions.

#### Procedure

Each participant was tested individually in a quiet room. All experiments were administered the same day with a pause after each condition.

In the discrete condition, both digit (color) stimuli and pseudo-words were displayed singly on a PC screen controlled by DMDX software (Forster and Forster, 2003) according to the following trial sequence: 15 ms acoustic tone, 400 ms blank field, 250 ms fixation cross, stimulus onset. The stimulus disappeared at pronunciation onset or after 4000 ms. Stimuli appeared in a pseudo-randomized fixed order in each block. The child was instructed to name the digit (or color name) or to read the pseudo-word aloud as fast and accurately as possible. Reaction time was measured and the whole utterance was digitally recorded.

In the multiple condition, a total of four sheets (two for each of the types of stimulus) were presented to the participant. The child was instructed to name the items aloud as fast and accurately as possible, progressing row-by-row and from left to right. The total time to complete the task was measured with a stopwatch and the errors were noted.

A short practice preceded task execution, separately for the different conditions. The order of conditions (discrete, multiple) as well as the order of type of stimulus (color, digits; five- or seven-letter pseudo-words) was counterbalanced across participants.

#### Data analysis

In the discrete condition, naming or reading times per item were the time between the onset of the stimulus and the offset of the vocal response (manually detected by means of Check Vocal software; Protopapas, 2007).

In the multiple condition, total naming times per lists were computed and divided by the number of stimuli in the arrays (100) to obtain a measure of naming time per item.

Preliminary analyses indicated some moderate tendency of the distribution of time scores to be skewed as often reported for this type of measures. In particular, the discrete digit naming condition deviated appreciably from normal distribution (Chi-Square goodness-of-fit test = 9.03, *p* < 0.05) although data from the other conditions did not deviate significantly (all *p*s > 0.05). Thus, inverse transformations for all measures were used, i.e., number of items/s. Normality tests indicated that none of these scores deviated from the normal distribution (all *p*s > 0.05 according to the Chi-squared goodness-of-fit test). So, this measure was adopted for all conditions.

Z scores were computed separately for digits and colors based on the group condition means and SDs. This was done separately for the discrete and multiple conditions. To obtain a single measure for pseudo-word reading performance in the discrete conditions, data for five- and seven-letter pseudo-words were collapsed. Z scores were computed based on the group condition means and SDs and averaged to obtain a single z score for pseudo-words.

To summarize, the final time measures entered in the analyses were: text reading (MT reading test), discrete pseudo-word reading, multiple digit (or color) RAN and discrete digit (or color) naming.

To test the influence of predictors on reading fluency in text reading we used commonality analysis, a method of variance partitioning designed to identify proportions of variance in the dependent variable that can be attributed uniquely to each of the independent variables, and proportions of variance that are attributed to various combinations of independent variables (Pedhazur, 1982; Nimon, 2010). To test our hypothesis that fluency in text reading can be effectively predicted by orthographic decoding and integration of reading sub-components, we first ran an analysis using only discrete pseudo-word reading and multiple digit (or color) RAN as predictors. Then we added the additional predictor “discrete digit (or color) naming” to see whether there was an increase in the explanatory power of the analysis.

### Results

Table 1 presents the matrix of inter correlations between all predictors and the dependent variable, i.e., fluency in text reading. A 0.003 *p* level (based on Bonferroni correction for multiple comparisons) was adopted. An inspection of the table identifies a number of major results:

– digit and color conditions are significantly correlated both in the case of multiple RAN and discrete naming but the latter correlation (between two typically ASAP tasks) is appreciably higher than on the RAN conditions;
– Performance on the multiple digit RAN task is correlated with text reading (the correlation for the multiple color RAN condition fails short of significance after correction for multiple comparisons);
– discrete digit or color naming show very low and insignificant correlations with text reading.
– multiple RAN (whether digits or colors) and discrete naming (digits or colors) are insignificantly correlated;
– multiple digit RAN is significantly correlated with discrete pseudo-word reading (the correlation for the multiple color RAN task fails short of significance after correction for multiple comparisons);
– finally, discrete naming (whether digits or colors) and discrete pseudo-word reading are significantly correlated.

**Table 1:** **Matrix of correlations between all predictors and the dependent variable, i.e., speed in text reading (MT test) for the group of proficient readers**.

	**Text reading**	**Multiple RAN (digits)**	**Multiple RAN (colors)**	**Discrete naming (digits)**	**Discrete naming (colors)**	**Discrete pseudo-word reading**
Text reading (speed)	–	0.49^*^	0.36	0.03	0.04	0.53^*^
Multiple RAN (digits)		–	0.49^*^	0.16	0.19	0.38^*^
Multiple RAN (colors)			–	0.16	0.28	0.33
Discrete naming (digits)				–	0.81^*^	0.65^*^
Discrete naming (colors)					–	0.56^*^
Discrete Pseudo-word reading						–

* p < 0.003.

Tables 2A,B presents the results of the multiple regression analysis using the digit conditions. Table 2A reports the commonality coefficients for the multiple digit RAN and discrete pseudo-word reading variables. As to the percentage of variance explained (see the rightmost column in Table 2A), the unique contributions of the “multiple digit RAN” (27.24%) and “discrete pseudo-word reading” (34.61%) variables are present as well as the commonality between the two predictors (38.15%).

Table 2**(A) Commonality coefficients and percentage of explained variance for predictors of text reading (“Multiple RAN” and “Discrete pseudo-word reading”): proficient readers (MODEL 1). (B) Unique and common contributions of “Multiple RAN” and “Discrete pseudo-word reading” to fluency measure: proficient readers (MODEL 1)**.

**Table:** 

**A**		
**Variables**	**Coefficient**	**Percent**
Unique to “Multiple RAN”	0.10	27
Unique to “Discrete pseudo-word reading”	0.13	35
Common to “Multiple RAN” and “Discrete Pseudo-word reading”	0.14	38
Total	0.37	100

**Table:** 

**B**									
	***R***	***R*^2^**	***R*^2^ adj**.	**ß st**.	***p***	**Unique**	**Common**	**Total**	**% of *R*^2^ (*r*^2^_s_)**
Model	0.61	0.37	0.34						
Multiple RAN				0.35	0.015	0.10	0.14	0.24	65.4%
Discrete pseudo-word reading				0.39	0.006	0.13	0.14	0.27	72.8%

Adj., adjusted; St., standardized; Unique, predictor's unique effect; Common, predictor's common effects; Total, Unique + Common; % of R^2^, Total/R^2^.

Unique and common contributions are summarized in Table 2B along with other parameters of the analysis, including the total variance explained by the model (37%) and the standardized β coefficients (and their significance values). For the sake of presentation we refer to this model as “*Model 1*”. The last column of the table reports the percentage of variance explained by the two factors considered (due to the presence of the common variance of the two factors the sum of the values exceeds 100%). By and large, results for the color conditions (“*Model 1 color*”) are consistent with those for the digit conditions (see Supplementary Materials).

Tables 3A,B presents “*Model 2*,” i.e., the commonality coefficients when the “discrete digit naming” variable is added as a predictor to the multiple regression analysis. Unique and common contributions are summarized in Table 3B along with the other parameters of the analysis, including the total variance explained by the model and the standardized β coefficients. Note that the total variance explained by “*Model 2*” increases substantially with respect to “*Model 1*,” passing from 37 to 52%. This increase is due to the influence of the “discrete naming” variable; specifically, the effect of this variable is suppressive with regard to the influence of the “discrete pseudo-word reading” variable (coefficient: −0.14 corresponding to 27.52% of explained variance, Table 3A). Again, results for the color conditions were similar (see Supplementary Materials).

Table 3**(A) Commonality coefficients and percentage of explained variance for predictors of text reading (“Multiple RAN,” “Discrete digit naming” and “Discrete pseudo-word reading”): proficient readers (MODEL 2). (B) Unique and common contributions of Multiple RAN, Discrete Naming and Discrete pseudo-word reading to fluency measure: proficient readers (MODEL 2)**.

**Table:** 

**A**		
**Variables**	**Coefficient**	**Percent**
Unique to “Multiple RAN”	0.07	13
Unique to “Discrete digit naming”	0.15	28
Unique to “Discrete pseudo-word reading”	0.27	52
Common to “Multiple RAN” and “Discrete digit naming”	0.03	6
Common to “Multiple RAN” and “Discrete pseudo-word reading”	0.18	34
Common to “Discrete digit naming” and “Discrete pseudo-word reading”	−0.14	−27
Common to “Multiple RAN,” “Discrete digit naming” and “Discrete pseudo-word reading”	−0.03	−6
Total	0.52	100

**Table:** 

**B**									
	***R***	***R*^2^**	***R*^2^ adj**.	**ß st**.	***p***	**Unique**	**Common**	**Total**	**% of *R*^2^ (*r*^2^_s_)**
Model	0.72	0.52	0.48						
Multiple RAN				0.29	0.023	0.07	0.18	0.24	47.1%
Discrete digit naming				−0.51	0.001	0.15	−0.14	0.00	0.1%
Discrete pseudo-word reading				0.75	0.000	0.27	0.00	0.27	52.4%

*Adj., adjusted; St., standardized; Unique, predictor's unique effect; Common, predictor's common effects; Total, Unique + Common; % of R^2^, Total/R^2^*.

### Discussion

Both basic factors, i.e., orthographic decoding and integration of reading sub-components contributed significantly to the overall prediction of text reading fluency. Furthermore, the prediction was higher when discrete naming was added to the model than when only the two original factors were considered. The general pattern of findings was similar for the digit and color conditions indicating that is the variance common to these two sets of stimuli to carry the relationship.

As to the orthographic decoding factor, performance in discrete pseudo-word reading exerted a large unique influence in the analyses with both the two- and three-factor models (i.e., “*Models 1* and *2*”). We proposed that this factor marks the individual efficiency of the pre-lexical graphemic description of the letter string (Zoccolotti et al., 2008).

As to the integration of the reading sub-components factor, the presence of a unique contribution of multiple RAN confirms that RAN tasks capture a proportion of variance (coefficient 0.07; about 13% of explained variance in “*Model 2*,” Table 3A) which is different from that accounted for by orthographic processing. This is in keeping with the idea that the RAN paradigm captures a portion of variance related to the processing of multiple stimuli.

The two variables also exerted a substantial influence together. One might think that the degree of efficiency in dealing with orthographic analysis of a string of letters contributes to managing multiple stimuli. In this vein, the interaction between multiple naming and reading would change as a function of reading experience. There is some evidence that the correlation between RAN and reading increases with reading experience (Kirby et al., 2003). Furthermore, Protopapas et al. (2013) recently reported that the co-variance between reading and RAN is best expressed in terms of a reading-naming latent structure in younger children and in terms of a serial-discrete dimension in more experienced children.

**Figure 1:**
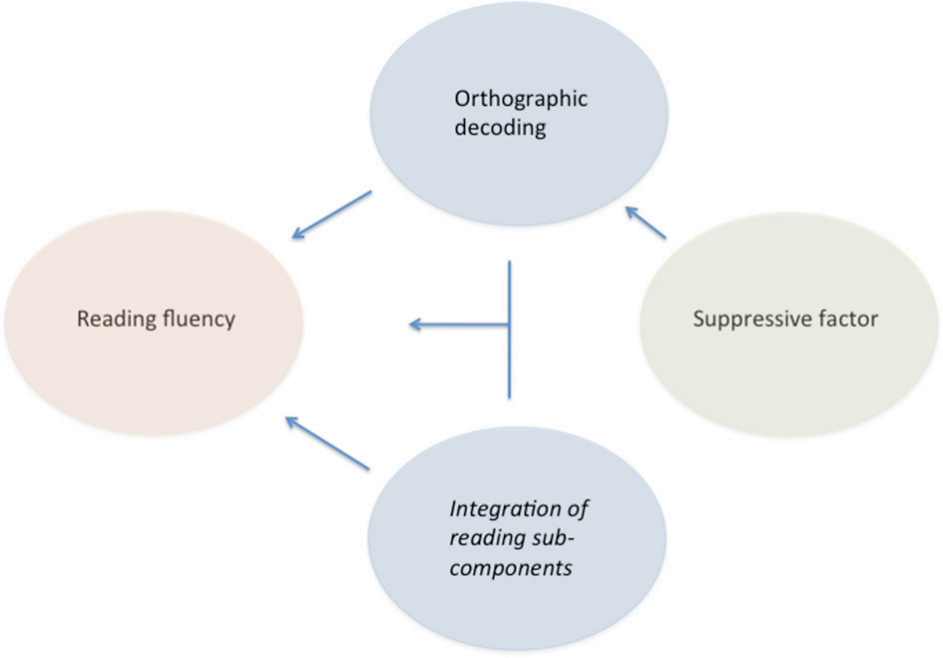
**Factors affecting individual differences in word fluency in typically developing readers**. Note that the suppressive factor exerts an effect on reading fluency only indirectly through the orthographic decoding but not through the integration of reading sub-components factor.

These relationships are schematized in Figure 1. Note that orthographic decoding and integration of reading sub-components influence reading fluency directly (both singly and interaction between each other). An indirect influence is also presented in the figure; indeed, the discrete digit naming variable exerted a suppressive effect selectively on discrete pseudo-word reading (but not on multiple RAN). A suppressor variable is one that is not directly correlated with the dependent variable but acts indirectly through another predictor(s) (note the insignificant correlation in Table 1 between reading fluency and digit or color naming). When added to the model the suppressive factor allows for a better overall prediction by accounting for some irrelevant variance in the predictor variables resulting in an increase of the relationship between the predictors and the outcome. This was clearly the case when we passed from the two-variable (“*Model 1*”) to the three-variable analysis (“*Model 2*”) and obtained an increase in explanatory power (from 37 to 52%; from 31 to 43%, in the case of the color conditions).

The idea that naming isolated non-orthographic items can have a suppressive effect in accounting for individual differences in reading was first conceived by Logan et al. (2011) and later supported by Protopapas et al.'s (2013) findings. Furthermore, Logan and Schatschneider (2014) recently re-analyzed seven different studies and confirmed that isolated naming acts as a suppressor variable in the relation of serial naming with reading. The present results are in part consistent with these previous studies and in part different. In considering the different outcomes it must be noted that Logan et al. (2011) only examined tasks with non-orthographic stimuli. By contrast, we observed (“*Model 2*”) that the suppressive effect of the discrete digit naming variable was mostly on discrete pseudo-word reading (i.e., −27.52%) and was not detected directly on multiple RAN (a very small suppressive effect, i.e., −6.58%, was present on the variance common to discrete pseudo-word reading and RAN).

This pattern of findings can be used to try to understand the nature of the suppressive effect. As this was unknown until recently, only tentative proposals can be advanced. For example, as their data indicated a suppressive effect over RAN, Logan et al. (2011) originally proposed that eye movements and parafoveal processing should be examined as possible targets of future research to explain the suppressive effect (for similar considerations see Logan and Schatschneider, 2014). In the present study the suppressive effect of discrete naming was on discrete pseudo-word reading, i.e., a condition with single, foveally presented orthographic stimuli; thus, Logan and co-workers' proposal would not easily fit the present data.

Another possibility is that what is being suppressed is naming speed. Within this idea, discrete naming taps the efficiency in the retrieval of phonological labels (whether directly linked to arbitrary mappings as in the case of digits or through semantic activation as in the case of colors). Efficient naming of discrete digit (or color) with ASAP instructions shares variance with discrete pseudo-word reading as it has in common the requirement to quickly retrieve and activate a phonological label after stimulus onset. By contrast, discrete naming is not directly related to reading fluency; thus, efficient phonological retrieval is not the reason that pseudoword decoding is related to text fluency. As stated above, one may envisage that the key factor for pseudo-word reading to predict reading is that it captures variance related to the processing of a (relatively long) string of graphemes.

Yet another, more general, alternative is that the portion of variance of the discrete naming variable which generates the suppressive effect is the requirement for a fast response to an externally triggered imperative stimulus under ASAP instructions. Indeed, this requirement is common to the discrete naming and discrete pseudo-word reading while it is not shared by discrete naming and text reading fluency (where is the subject to set his/her own pacing in proceeding through the text). In this vein, what is being suppressed by the discrete naming of colors/digits can be seen as expressing individual “cognitive speed.” While this term may appear overly general, Faust et al. (1999) specify rather specific conditions to define this dimension and we refer to their formulation here. Accordingly, cognitive speed expresses the commonality that is present across many speeded decision tasks and that indicates the overall information processing rate characteristic of a given individual. Typical within this frame are studies of the general slowing observed with aging (e.g., Cerella, 1990). Faust et al. (1999) showed that commonality emerges quite clearly in factor analyses of tasks requiring a response under time constraints, i.e., in conditions in which the subject must respond ASAP to an external stimulus that triggers the response. In this perspective, cognitive speed marks the individual information processing rate across many tasks and modalities.

The present data are consistent with this interpretation although they cannot prove it. For this reason in the scheme of Figure 1 we use the neutral term “suppressive factor,” even though we feel that the “cognitive speed” factor represents a coherent and comprehensive framework to interpret it. Further comments on the suppressive factor will be advanced in the general discussion.

## Study 2: predicting reading fluency in dyslexic children

The development of reading progresses from early acquisition of orthographic decoding to a later ability to effectively integrate decoding with the other sub-components of reading. In the words of Buswell (1921): “*An immature reader … tends to keep the eye and voice very close together, in many cases not moving the eye from a word until the voice has pronounced it. Reading of this type becomes little more than a series of spoken words because there is no opportunity to anticipate the meaning in large units*.”

This pattern of reading was confirmed experimentally by Protopapas et al. (2013) examining discrete and serial naming of digits, objects and words in Greek second and sixth graders. Discrete and serial word reading correlated very highly in Grade 2 but only moderately in Grade 6. Protopapas et al. (2013) concluded that “*word fluency tasks in Grade 2 are apparently accomplished largely as a series of isolated individual word naming trials even though multiple individual letters in each word may be processed in parallel. In contrast, specifically serial procedures are applied in Grade 6, presumably via simultaneous processing of multiple individual words at successive levels*.”

As young readers dyslexic children may be expected to process stimuli in an isolated fashion, as indicated by their smaller eye-voice lead (Buswell, 1921; Fairbanks, 1937; De Luca et al., 2013). According to the proposed model, this can be captured in part from their (defective) performance on the multiple RAN tasks; furthermore, one may believe that the orthographic decoding factor is particularly important in these children as compared to typically developing readers. This may be expressed as greater weight of this factor in the prediction or, alternatively, as a dominant role of this factor over and above the moderating influence of the discrete naming variable.

### Materials and methods

#### Participants

Twenty-five children with dyslexia (14 males and 11 females; mean age 11.8 ± 0.8 years) participated in the experiment. Children were comparable for age and gender to the typically developing readers in Study 1. To assess non-verbal IQ levels, we used the scores obtained by 12 children with dyslexia on Raven's Colored Progressive Matrices. All children scored well within the normal limits according to Italian norms (Pruneti et al., 1996). Mean raw score was 27.3 ± 2.6; mean z score was −0.66 ± 0.62. Wechsler Intelligence Scale for Children (WISC) data were available for the other 13 children with dyslexia; scores were well within the normal range for both performance and verbal subscales (mean total score 96.2 ± 10.1). All participants had normal or corrected-to-normal visual acuity.

The children with dyslexia scored at least 1.65 standard deviations below the norm for either speed or accuracy on the MT Reading test (Cornoldi and Colpo, 1995). As for raw data, the average reading time per syllable was 0.51 s (*SD* = 0.17), and mean number of errors was 21.7 (*SD* = 9.1). Based on normative values (Cornoldi and Colpo, 1995), mean z scores were −2.50 and −2.97, for reading time and accuracy respectively.

As for typically developing children, an inverse transformation was applied to the data so that item/s was considered in the statistical analyses. So, reading speed at the MT test (in terms of word/s) was the dependent measure to estimate text reading fluency.

#### Experimental conditions procedure

All measures were computed as described above.

As for reading/naming time measures, the pseudo-word reading condition deviated appreciably from normal distribution (Chi-Square goodness-of-fit test = 12.78, *p* < 0.001) while data from all the other conditions did not deviate significantly (all *p*s > 0.05). Thus, inverse transformations for all measures were used, i.e., number of items/s, as for typically developing children. Normality tests indicated that none of these scores deviated from the normal distribution (all *p*s > 0.05 according to the Chi-squared goodness-of-fit test).

#### Data analysis

As described above.

### Results

Table 4 presents the matrix of inter correlations between all predictors and the dependent variable (i.e., speed in text reading), for the sample of children with dyslexia. A 0.05 significance level was adopted; as we were interested in comparing this pattern of results with those of typically developing readers no correction for multiple comparisons was considered in this case.

**Table 4:** **Matrix of correlation between all predictors and the dependent variable (text reading fluency), speed in text reading (MT test) for the group of dyslexic readers**.

	**Text reading**	**Multiple RAN (digits)**	**Multiple RAN (colors)**	**Discrete naming (digits)**	**Discrete naming (colors)**	**Discrete pseudo-word reading**
Text reading (speed)	–	0.66^*^	0.42^*^	0.56^*^	0.54^*^	0.78^*^
Multiple RAN (digits)		–	0.70^*^	0.42^*^	0.36	0.55^*^
Multiple RAN (colors)			–	0.33	0.25	0.34
Discrete naming (digits)				–	0.86^*^	0.69^*^
Discrete naming (colors)					–	0.62^*^
Discrete Pseudo-word reading						–

* *p* < 0.05.

The general pattern of correlations is similar to that observed with typically developing children. One main difference emerges: discrete naming (both digits and colors) is correlated with text reading. This is at variance with what occurs for typically developing readers where no correlation was detected.

Table 5A presents the commonality coefficients for the multiple RAN and discrete pseudo-word reading variables for the dyslexic children using the digit conditions. There is a detectable unique contribution of the multiple RAN variable (10.16% of explained variance). The unique contribution of the discrete pseudo-word reading variable is large (36.41%). Finally, the two predictors share 53.43% of the variance. Unique and common contributions of the two variables are summarized in Table 5B along with other parameters of the analysis, including the variance explained by the model and the standardized β coefficients. Note that the total variance explained by the model (referred to as “*Model 3*”) is high (69%). Results of the color conditions are again similar (see Supplementary Materials).

Table 5**(A) Commonality coefficients and percentage of explained variance for predictors of text reading (“Multiple RAN” and “Discrete pseudo-word reading”): dyslexic readers (MODEL 3). (B) Unique and common contributions of “Multiple RAN” and “Discrete pseudo-word reading” to fluency measure: dyslexic readers (MODEL 3)**.

**Table:** 

**A**		
**Variables**	**Coefficient**	**Percent**
Unique to “Multiple RAN”	0.07	10
Unique to “Discrete pseudo-word reading”	0.25	36
Common to “Multiple RAN” and “Discrete pseudo-word reading”	0.37	54
Total	0.69	100

**Table:** 

**B**									
	***R***	***R*^2^**	***R*^2^ adj**.	**ß st**.	***p***	**Unique**	**Common**	**Total**	**% of *R*^2^ (*r*^2^_s_)**
Model	0.83	0.69	0.66						
Multiple RAN				0.32	0.036	0.07	0.37	0.44	63.6%
Discrete pseudo-word reading				0.61	0.000	0.25	0.37	0.62	89.8%

Adj., adjusted; St., standardized; Unique, predictor's unique effect; Common, predictor's common effects; Total, Unique + Common; % of R^2^, Total/R^2^.

An additional multiple regression was carried out by adding the discrete digit naming predictor. The results of this analysis are presented in Tables 6A,B. Notably, the proportion of explained variance was the same after adding this variable (69%; “*Model 4*”). In the analysis the discrete digit naming variable shares some variance with the multiple RAN and pseudo-word reading variables but does not exert a suppressive effect (as in the sample of typically developing readers). The parallel results for the color conditions are reported Supplementary Materials.

Table 6**(A)Commonality coefficients and percentage of explained variance for predictors of text reading (“Multiple RAN,” “Discrete digit naming” and “Discrete pseudo-word reading”): dyslexic readers (MODEL 4). (B)Unique and common contributions of Multiple RAN, Discrete Naming and Discrete pseudo-word reading to fluency measure: dyslexic readers (MODEL 4)**.

**Table:** 

**A**		
**Variables**	**Coefficient**	**Percent**
Unique to “Multiple RAN”	0.07	10
Unique to “Discrete digit naming”	0.00	0
Unique to “Discrete pseudo-word reading”	0.16	23
Common to “Multiple RAN” and “Discrete digit naming”	0.00	0
Common to “Multiple RAN” and “Discrete pseudo-word reading”	0.15	22
Common to “Discrete digit naming” and “Discrete pseudo-word reading”	0.10	14
Common to “Multiple RAN”, “Discrete digit naming” and “Discrete pseudo-word reading”	0.22	31
Total	0.69	100

**Table:** 

**B**									
	***R***	***R*^2^**	***R*^2^ adj**.	**ß st**.	***p***	**Unique**	**Common**	**Total**	**% of *R*^2^ (*r*^2^_s_)**
Model	0.83	0.69	0.65						
Multiple RAN				0.32	0.041	0.07	0.37	0.44	63.6%
Discrete digit naming				0.002	0.915	0.00	0.31	0.31	45.0%
Discrete pseudo-word reading				0.60	0.004	0.16	0.47	0.62	89.8%

Adj., adjusted; St., standardized; Unique, predictor's unique effect; Common, predictor's common effects; Total, Unique + Common; % of R^2^, Total/R^2^.

### Discussion

In the case of children with dyslexia, the model with only two predictors i.e., “multiple RAN” and “discrete pseudo-word reading,” accounts for a large proportion of variance (69%) and no increase in explanatory power is obtained by adding the corresponding discrete naming variable. A note of caution in interpreting these data is in order given the relatively small sample size of dyslexic children, particularly considering the type of statistical analyses. This suggests the importance that the pattern of results be replicated in a different, larger sample, before definite conclusions be drawn. At any rate, results similar to those obtained considering the digit conditions were found using the color conditions. This finding points to the stability of the pattern observed at least within the sample examined.

Notably, the general structure of the model is similar to that of typically developing readers (as schematized in Figure 1). In the case of children with dyslexia, however, no suppressive effect of the discrete digit (or color) naming variable was detected when this was added to the model. Thus, it appears that for these children discrete pseudo-word reading performance is so heavily loaded with orthographic decoding that no additional power can be obtained by considering the moderating effect of discrete naming, or individual “cognitive speed” as proposed above.

## General discussion

To predict individual differences in text reading fluency in typically developing and dyslexic readers, we chose to evaluate factors that, based on previous research, clearly distinguished children with and without a reading deficit. We reasoned that the two selected tasks would selectively measure two different basic processes of reading fluency, i.e., the ability of the child to process a letter string and the ability to integrate this processing with on-going analysis of the text. For the time being, we have purposely ignored all higher-level linguistic processes, such as activation of lexical and semantic information and on-going syntactic processing, to determine how much individual reading rate depends on basic reading processing.

### Predicting speed in reading meaningful texts

The main result of the study is that the ability to decode letter strings (measured by the pseudo-word reading variable) and the ability to integrate the various sub-components at work in reading (measured by the RAN variable) jointly allow accounting for a sizeable amount of variance in reading fluency on meaningful texts. The reliability coefficient for our dependent measure, i.e., the MT Reading test time (Cornoldi and Colpo, 1995), is reported to be ca 0.90. Thus, the basic reading processes examined allow accounting for approximately two-thirds of the true variance in text fluency. This holds for both typically developing readers and dyslexic children although with a partially different pattern of predictors (see below). Notably, this high prediction occurs without considering higher level linguistic processes, which involve the activation of lexical, semantic and contextual information.

Below we discuss some specific, and partially open, questions related to the variables considered in the study; in the last section we speculate on the advantage of modeling reading deficits based on proximal rather than distal causes.

### Pseudo-word reading

Orthographic decoding contributed importantly to the prediction of reading fluency. The pseudo-word reading task putatively captures the ability to process a letter string and produce an appropriate phonological output. In the introduction, we presented evidence that children with dyslexia show a selective deficit when they have to deal with a letter string presented visually, the deficit being very similar whether the stimulus is a word or a pseudo-word (Zoccolotti et al., 2008; De Luca et al., 2010; Marinelli et al., 2011). We proposed that this deficit marks a pre-lexical impairment in forming a graphemic description of the stimulus, i.e., a deficit in the abstract representation of a letter string (Zoccolotti et al., 2008). In neural terms, the LCD model proposes that this ability rests on the output of a hierarchy of detectors tuned to increasingly larger and more complex word fragments (Dehaene et al., 2005). In this hypothesis the underlying factor refers essentially to visual perception.

Alternative hypotheses can also be considered to interpret this ability. One idea is that the phonological component of the processing is essential for generating the difference between dyslexic and control readers and in mediating the relationship with reading. Against a strict phonological interpretation, it has been shown that dyslexic readers' deficit is selective for the visual modality and the same stimuli presented acoustically are responded to flawlessly (Marinelli et al., 2011). Furthermore, clear deficits are present also when children have to process strings of consonants in tasks that minimize the influence of phonological activation (i.e., visual span paradigm; Bosse et al., 2007; Valdois et al., 2012). In the same vein, we recently completed a lexical decision experiment in which we used as foils either pronounceable pseudo-words (such as DASU) or unpronounceable non-words made of consonants (such as RNGM). Group differences in responses to words, pseudo-words and non-words were all accounted for by the same (letter-string) global factor indicating that pronounceability of the foil was not critical in mediating the deficit of dyslexic children (Marinelli et al., under revision). A more advanced hypothesis is that the binding between orthographic and phonological information is crucial in generating the dyslexic deficit (Ziegler et al., 2010a; van den Broeck and Geudens, 2012). Some recent neuroimaging evidence points in this direction. In a fMRI study, van der Mark et al. (2011) detected a significant disruption of the functional connectivity between the VWFA and left inferior frontal and left inferior parietal language areas in children with dyslexia. Therefore, the possibility must be considered that the critical underlying factor in the pseudo-word reading task is the need to connect a string of graphemes to the corresponding phonological output.

The possibility should also be considered that lexical activation contributes to performance of the pseudo-word reading task. On the whole, this hypothesis seems unlikely on several grounds. It has been proposed that pseudo-words may generate lexical effects or that parts of pseudo-words may be recognized holistically (e.g., Moll et al., 2009). However, this generally occurs under very specific conditions, such as when they are presented intermingled with words, but this did not occur in the present experiment. Furthermore, lexical attempts at reading pseudo-words are much more frequent among children learning to read an irregular orthography such as English than a regular orthography such as German (e.g., Wimmer and Goswami, 1994).

Overall, orthographic decoding plays an important role in the prediction of fluency in reading a text in a regular orthography such as Italian. Whether this performance essentially marks the efficiency of the graphemic processor of letter strings or of a mechanism binding the output of this processor to phonological processing is beyond the aims of the present study and is a question open to future research.

### RAN

The finding that performance on the RAN tasks actually predicts reading fluency confirms much previous research (Wolf and Bowers, 1999; Wolf et al., 2000). Based on evidence summarized in the Introduction, we considered that RAN tasks selectively capture the ability to integrate the various sub-components necessary for effective reading but exclude orthographic decoding. Critical in this perspective is the finding that RAN correlates with reading only if the task requires serial processing and active production of specific names (Georgiou et al., 2013), as occurs in reading. The present results indicate that RAN tasks account for a sizeable amount of variance (more than 10% in both groups of children) over and above that accounted for by orthographic decoding, and that accounted in common by the two factors. This finding confirms previous observations by Protopapas et al. (2013) who found RAN to contribute unique variance over and above discrete word reading at least in 6 grade children. Overall, the RAN tasks capture individual variability linked to the ability to deal with multiple targets; note that this variability cannot be explained in terms of processing the same stimuli when presented in a discrete format (Georgiou et al., 2013; present data).

It is not clear at present whether these individual differences can be ascribed to a single identifiable mechanism. One hypothesis proposes that slowness in RAN tasks depends on a multiple, or domain-general, temporal processing deficit in dyslexic children (Farmer and Klein, 1995). However, a systematic check of this hypothesis failed to reveal any indication that a deficit in temporal processing *per se* underlies the reading deficit of dyslexic individuals (Chiappe et al., 2002). Alternatively, one can speculate that individual differences in the fluency to deal with multiple visual stimuli, such as digits or color, with the aim of naming them rest on a more specific skill. At least in part, this represents an individual trait present prior to school experience as it has been found that performance on RAN tasks at a pre-school stage significantly predicts later efficiency in reading (e.g., Bishop, 2003), However, this does not exclude that efficiency in RAN tasks is progressively tuned through reading itself (e.g., Torgesen et al., 1994). In fact, through reading training, children get much experience in integrating target identification with visual scanning, parafoveal pre-analysis and pronunciation. Thus, when we examine individual RAN speed in children who already attended school for a number of years, we measure a skill that has had received partial reinforcement from reading experience itself. In support of this view, the distinction between single-multiple stimuli processing becomes prominent in modulating the relationship with reading only after a number of years of schooling (Protopapas et al., 2013). Furthermore, while RAN tasks are correlated with reading across very different ages, the size of this correlation increases with reading experience (Kirby et al., 2003). Thus, the link in Figure 1 between “orthographic decoding” and “integration of reading sub-components” sketches a relationship between the two factors that is bidirectional and may presumably change with age and reading experience.

In the same vein, note that a much more complex model could be proposed following the (not unlikely) view that reading experience affects fluency, and fluency may affect both integration and decoding. Feedback links should integrate the model and different analyses may contribute to evaluating the direction and weight of each influence; however, we see the present study only as a first step in modeling individual variations in reading fluency in Italian typically developing readers and dyslexic children.

### Suppressive factor

The performance on the discrete digit or color naming task contributed as a third factor, and in a suppressive manner, to the prediction of reading fluency in typically developing but not in dyslexic readers. Above, we tentatively discussed a few alternative interpretations. Admittedly, the present data do not allow to persuasively select between a naming speed and a cognitive speed interpretation and only speculative considerations can be advanced at this point. However, as stated in the comments of study 1, cognitive speed seems to provide a theoretically sound interpretation and one that is potentially worth of further research.

Faust et al. (1999) define cognitive speed as the overall information processing rate characterizing a given individual across a variety of tasks (Faust et al., 1999). Indeed, in conditions with ASAP instructions the time measures of performance (RTs) on different tasks are always highly correlated. Due to this very large co-variation, if a standard factor analysis is applied to the data a single factor accounts for a large proportion of individual variability (Faust et al., 1999). At first glance, this finding contrasts with the well-known fact that RTs are particularly sensitive in picking up differences due to experimental manipulations. See, for instance, the effects of psycholinguistic variables (such as word frequency, orthographic neighbors, etc) on word recognition that typically reveal significant effects with differences of a few milliseconds. When testing the effects of experimental manipulations, this large co-variation is controlled for by the use of repeated measures designs (which essentially partial out the correlation between measures across experimental conditions). However, if one wants to examine individual differences (rather than the effects of experimental manipulations) one must face the fact that the time measures will all be highly correlated, particularly when the general format of task and response is kept constant (as in RT tasks in which the subject has to respond ASAP to an external imperative stimulus). In these cases, the presence of correlation will substantially modulate the relationships between the specific factors investigated. This represents a problem if, as in the present case, no correlation is actually expected between reading fluency and cognitive speed *per se* (as shown by Bonifacci and Snowling, 2008); however, measures of cognitive speed will correlate with other predictors provided that they share the general task format which may indeed be more important than the specific type of stimuli. In this vein, it is interesting that the discrete digit/color naming task has a large suppressive effect on pseudo-word reading with which it shares a general format (i.e., an ASAP response), but has no detectable effect on the RAN tasks with which it shares the type of stimulus (digits or colors) but not the general format.

This framework may help placing the lack of an effect in dyslexic children. Based on readers' data, we should expect the cognitive speed factor to modulate pseudoword reading. However, this influence was not significant because the dramatic slowness of dyslexic children in orthographic decoding also implies huge individual differences at this level and dominates over the cognitive speed factor.

In the introduction and above we have cited evidence indicating that a global factor marks individual performance in speeded reading tasks and effectively discriminates between dyslexic and typically developing readers. However, the global factor that marks dyslexics' performance (Zoccolotti et al., 2008) and the cognitive speed factor described by Faust et al. (1999) are clearly distinct constructs. Dyslexic children are slow across many tasks, but only if they require the processing of orthographic strings. By contrast, according to Faust et al. cognitive speed refers to a more general construct, spanning across different stimuli and modalities.

### Modeling individual differences in reading fluency: proximal vs. distal causes

The present approach should be distinguished from several previous attempts to predict individual reading performance (e.g., Torgesen et al., 1997; Muter and Snowling, 1998; Compton et al., 2001; Kirby et al., 2003; Ziegler et al., 2010b; Landerl et al., 2012; Warmington and Hulme, 2012). In these studies, the authors aimed to predict reading using a variety of cognitive measures but without explicitly attempting to make a componential analysis of reading behavior. Characteristically, a spectrum of linguistic, meta-phonological, visual and also RAN measures are used jointly to examine which predictor(s) is (are) more strongly related to the reading dependent measure.

One way to distinguish this approach from the present one is to see it as focusing on distal (as opposed to proximal) causes of behavior. Within a proximal approach (such as in the present study), reading behavior is described in terms of the building blocks of the reading processes (see further comments below). By contrast, a distal approach has the more ambitious goal of searching for the ultimate origin of normal and disordered behavior. Thus, predictors are considered as inherently independent causes of behavior and, as such, the presence of uni-directional links between putative causes and effects is an essential tenet of this approach. However, this assumption is problematic when using cognitive markers as distal “causes” of individual variability in reading.

This point has been often discussed in relation to phonological awareness. One popular view considers phonological awareness as a critical ability for the beginning of reading and defective phonological awareness as a possible cause of dyslexia (for a review see Melby-Lervag et al., 2012). In this vein, phonological awareness is a distal cause of reading behavior, exerting a unidirectional relationship. However, this assumption is questioned by the observation that the critical learning for the conscious awareness of phonemes actually occurs during schooling (Morais et al., 1979). The influence of school experience is particularly clear in studies comparing later-schooled children (i.e., children who start school 1 or 2 years after the usual age) with children matched for age but differing for school experience, and children matched for schooling but differing for age (Alcock et al., 2010; Cunningham and Carroll, 2011; for similar data on Italian children see Scalisi et al., 2013). Thus, phonological awareness may be seen more as a consequence than a cause of reading. More complex interpretations have been advanced that propose the presence of a reciprocal relationship between phonological awareness and reading (e.g., Perfetti et al., 1987). However, even if this were appropriate, it would seriously undermine the validity of using phonological awareness as a distal, unidirectional predictor of reading.

Although this question has been often discussed in relation to phonological awareness, it presumably also refers to other general cognitive predictors, such as vocabulary breadth or visual scanning. Indeed, the same argument may well apply to RAN even though the change in performance after the beginning of schooling is not as abrupt as in the case of phonological awareness tasks (e.g., Scalisi et al., 2013). As stated above, it seems reasonable to envisage that children tune their ability to integrate scanning, identify and name visual targets mostly through reading experience and it is with reading experience that individual differences in the fluidity of carrying out such complex behaviors come out most clearly (Kirby et al., 2003; Protopapas et al., 2013).

Overall, for a distal approach to be effective it is crucial that cognitive predictors be independent from the behavior to predict, i.e., that the direction of causality be unidirectional. However, this assumption seems very difficult to hold in view of the strict bidirectional relationship that most of the cognitive abilities (as phonological abilities and integrating skills) typically entered in prediction studies hold with reading.

Another critical characteristic of the distal approach in the case of reading and dyslexia is that the nature of the relationship between the cognitive measures and reading are usually left under-specified in terms of actual processes. One can imagine, for example, various ways in which low short-term memory, small vocabulary or inefficient ability to segment or blend phonemes can indeed affect the acquisition of reading. Yet, no explicit relationship is typically formulated as to which specific cognitive deficiency should produce which selective effect on reading. Put in other terms, predictors do not have a specific place in the architecture of reading.

In the present study, we viewed the predictions about reading from the perspective of proximal causes. According to some influential authors, this approach has its own autonomy even in the absence of a full description of the distal causes of dyslexia, although these will eventually need to be investigated (e.g., Jackson and Coltheart, 2001, 2002). In the proximal approach, it is not critical that expertise in orthographic decoding and integration of reading sub-components are progressively tuned throughout schooling and, more generally, with reading experience, i.e., that they are not fully independent causes exerting a unidirectional influence on reading fluency. What is crucial is to spell out the building blocks of the reading process and evaluate their individual and interactive influence on individual reading fluency. In this view, note that here we qualify RAN performance as a measure of a specific component of the reading process, namely the integration of reading sub-components (see Georgiou et al., 2013), not as a general cognitive predictor. There is a long tradition of studies based on the proximal approach, particularly stemming from the dual route model (Coltheart et al., 2001). Most often, they have dealt with the analysis of single case studies. Here, we propose that a proximal approach may help to re-think the correlational studies predicting individual variability in text reading.

Finally, a novel methodological element of the approach used in the present study is the homogeneity of the measures adopted. By focusing specifically on reading speed, we used the same measure (i.e., total reading time per item) across both independent and dependent variables. Most previous studies on the prediction of reading used mixed measures and included reading accuracy as a dependent variable even in cases in which speed measures were used as predictors (Logan and Schatschneider, 2014). In these cases, variations in the format of the measures used might have unknown effects on the pattern of relationships found.

### Conclusions

The results of the present study indicate that fluency in reading texts depends heavily on basic reading processes, i.e., the ability to decode letter strings and to integrate the various reading sub-components. This prediction occurs without considering the role of lexical, semantic and contextual information. Although these processes may also exert some influence, it seems that they can only complement the prediction in view of the large proportion of variance accounted for by basic reading processes. In typically developing readers, the prediction becomes more effective when the suppressive effect of stimulus-triggered naming speed under ASAP instructions is considered, suggesting a putative indirect role of individual cognitive speed.

### Conflict of interest statement

The authors declare that the research was conducted in the absence of any commercial or financial relationships that could be construed as a potential conflict of interest.

## Appendix

### Pseudo-words

acria; barta; carvo; cospa; curno; dribo; ersia; fergo; gorra; lispo; macca; natto; pesso; pocre; pucca; risbo; terpa; tuore; turra; vazio. aldirgo; ardesto; bilevio; candima; conzane; cunallo; dascone; finecia; guaspia; nattoga; pestora; podilla; rucchia; runazzo; tarenno; tembara; tigiala; tivarna; valtano; vamione.
