# Supplementary material for: Modeling individual differences in text reading fluency: a different pattern of predictors for typically developing and dyslexic readers
Source: Front Psychol. 2014 Nov 18;5:1374. doi: 10.3389/fpsyg.2014.01374 (PMC4235379; doi:10.3389/fpsyg.2014.01374)
Supplement: Supplementary file 1 [file DataSheet1.PDF]

Supplementary Materials

The appendix reports the same analyses as in the main text but run over colour stimuli. Thus, multiple RAN refers to the performance on multiple colours naming; discrete colour naming refers to the performance in naming discrete patches of colour.

Table A. *Unique and common contributions to fluency measure: proficient readers. The upper part of the table reports MODEL 1colour, i.e., the model based on the "Multiple RAN" and "Discrete pseudo-word reading" variables. The lower part of the table reports MODEL 2colour, i.e., the model based on the "Multiple RAN" and "Discrete pseudo-word reading" and "Discrete colour naming" variables.*

	R	R^2	R^2 adj	β st	p	Unique	Common	Total	% of R^2 (r_s^2)
**Model *1colour***	.56	.31	.28						
Multiple RAN				.35	.134	.04	.09	.13	41.79%
Discrete pseudo-word reading				.21	.002	.18	.09	.27	87.10%
**Model *2colour***	.65	.43	.38						
Multiple RAN				.26	.054	.06	.07	.13	30.44%
Discrete colour naming					.008	.12	-.12	.00	0.33%
Discrete pseudo-word reading				.67	.000	.29	-.02	.27	63.44%

Legend: Adj = adjusted; St = standardized; Unique = predictor's unique effect; Common = predictor's common effects; Total = Unique + Common; % of R^2 = Total/R^2

Table B. *Unique and common contributions to fluency measure: dyslexic readers. The upper part of the table reports MODEL 3colour, i.e., the model based on the "Multiple RAN" and "Discrete pseudo-word reading" variables. The lower part of the table reports MODEL 4colour, i.e., the model based on the "Multiple RAN" and "Discrete pseudo-word reading" and "Discrete colour naming" variables.*

	R	R2	R2 adj	β st	p	Unique	Common	Total	% of R2 (rs2)
**Model *3colour***	.81	.65	.62						
Multiple RAN				.18	.200	.03	.15	.18	27.73%
Discrete pseudo-word reading				.73	.000	.47	.15	.62	95.72%
**Model *4colour***	.81	.65	.60						
Multiple RAN				.17	.217	.03	.15	.18	27.62%
Discrete colour naming				.06	.701	.00	.29	.29	44.14%%
Discrete pseudo-word reading				.69	.001	.27	.35	.62	95.35%

Legend: Adj = adjusted; St = standardized; Unique = predictor's unique effect; Common = predictor's common effects; Total = Unique + Common; % of R^2 = Total/R^2
